# Supplementary material for: Phase separation of FSP1 promotes ferroptosis
Source: Nature. 2023 Jun 28;619(7969):371–7. doi: 10.1038/s41586-023-06255-6 (PMC10338336; doi:10.1038/s41586-023-06255-6)
Supplement: Supplementary file 1 — This file contains Supplementary Figs. 1 and 2. Supplementary Fig. 1: An example of the gating strategy for the lipid peroxidation assays using C11-BODIPY. Supplementary Fig. 2: Uncropped gel images. [file 41586_2023_6255_MOESM1_ESM.pdf]

nature portfolio

https://doi.org/10.1038/s41586-023-06255-6

# Supplementary information

# Phase separation of FSP1 promotes ferroptosis

In the format provided by the authors and unedited

Supplementary Figure

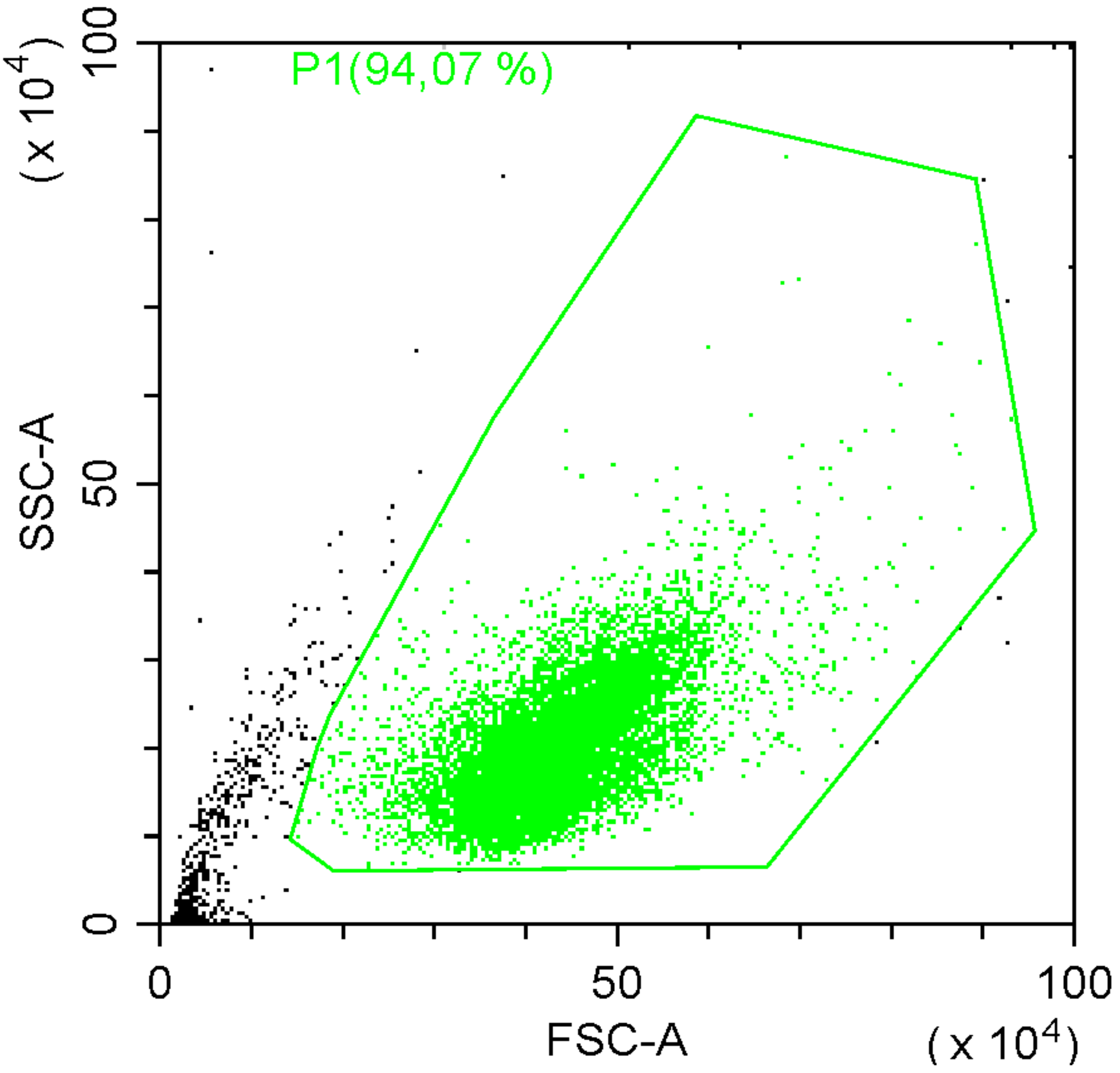


**Supplementary Fig.1**
An example of the gating strategy for lipid peroxidation assay using C11-BODIPY
Green area was considered as viable cells and analyzed.

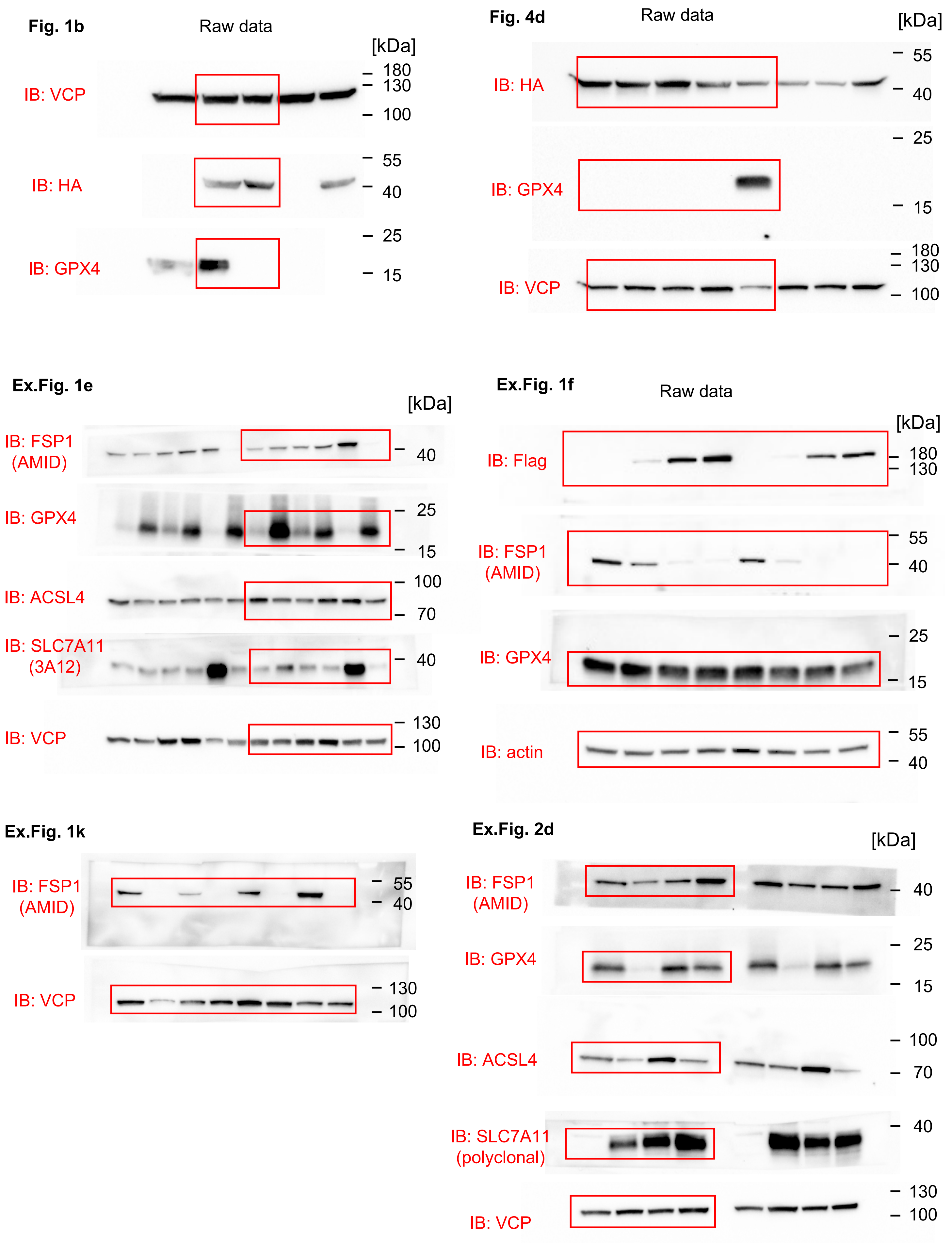


**Supplementary Fig.2**
Raw images of immunoblotting with a molecular marker were shown.
Red area were cropped for visualization.

**Ex.Fig. 2e**

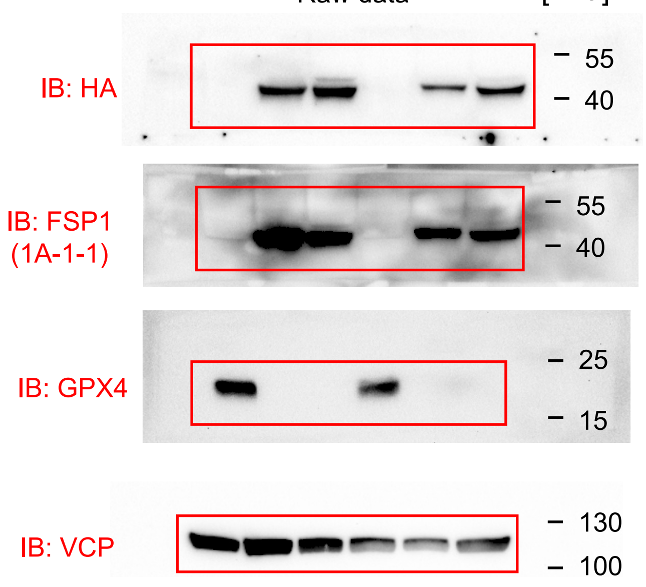


**Ex.Fig. 2k**

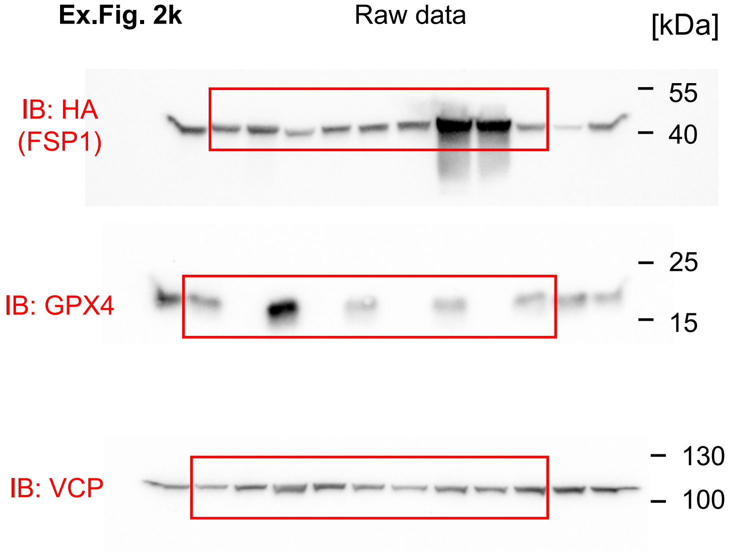


**Ex.Fig. 3l**

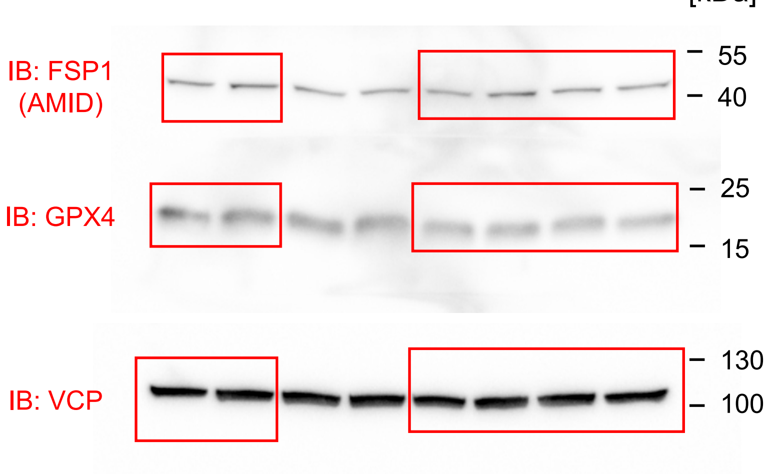


**Ex.Fig. 3m**

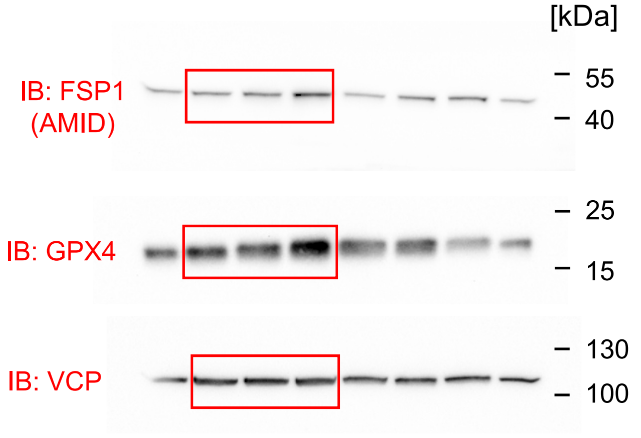


**Ex.Fig. 3n**

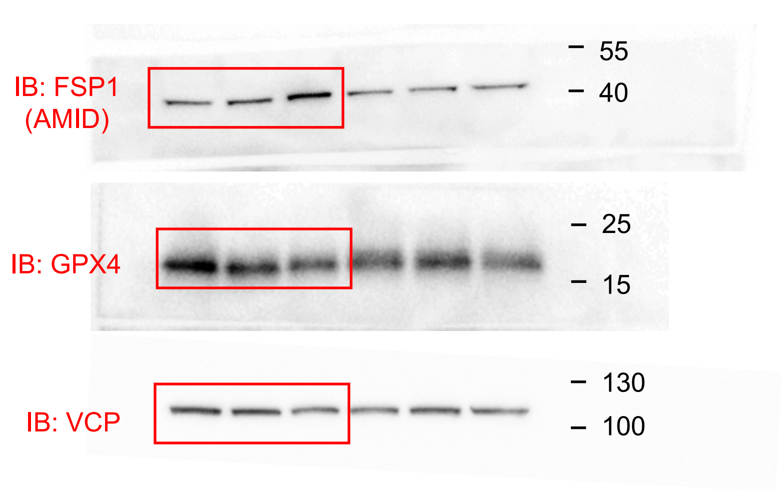


**Ex.Fig. 4e**

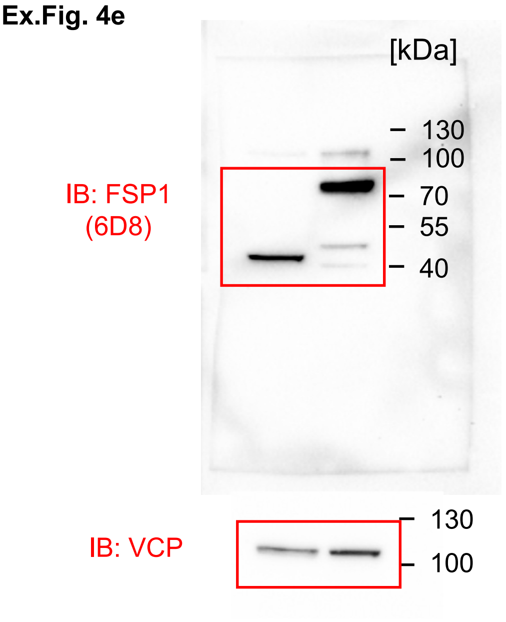


**Supplementary Fig.2 (continued)**

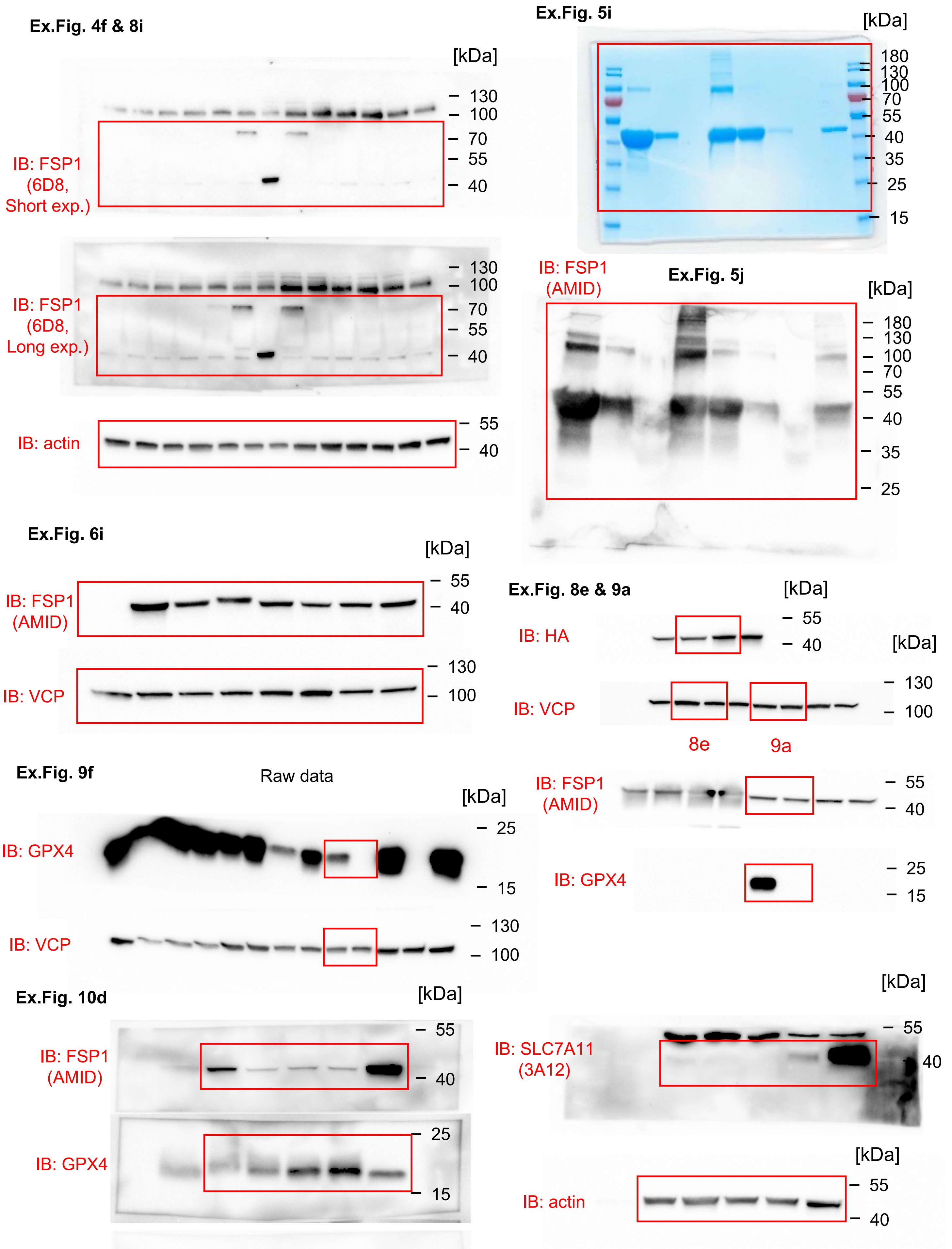


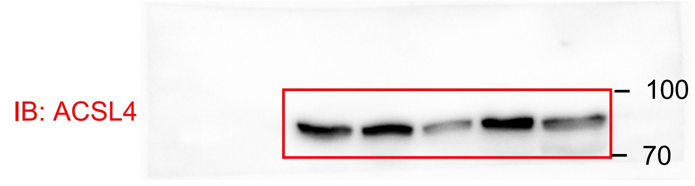


**Supplementary Fig.2 (continued)**
