# Supplementary material for: Phase separation of FSP1 promotes ferroptosis
Source: Nature. 2023 Jun 28;619(7969):371–7. doi: 10.1038/s41586-023-06255-6 (PMC10338336; doi:10.1038/s41586-023-06255-6)
Supplement: Supplementary file 2 — Reporting Summary [file 41586_2023_6255_MOESM2_ESM.pdf]

nature portfolio

Corresponding author(s): Marcus Conrad

Last updated by author(s): May 9, 2023

Reporting Summary

Nature Portfolio wishes to improve the reproducibility of the work that we publish. This form provides structure for consistency and transparency in reporting. For further information on Nature Portfolio policies, see our Editorial Policies and the Editorial Policy Checklist.

Statistics

For all statistical analyses, confirm that the following items are present in the figure legend, table legend, main text, or Methods section.

n/a	Confirmed	
☐	☒	The exact sample size (*n*) for each experimental group/condition, given as a discrete number and unit of measurement
☐	☒	A statement on whether measurements were taken from distinct samples or whether the same sample was measured repeatedly
☐	☒	The statistical test(s) used AND whether they are one- or two-sided<br>*Only common tests should be described solely by name; describe more complex techniques in the Methods section.*
☒	☐	A description of all covariates tested
☐	☒	A description of any assumptions or corrections, such as tests of normality and adjustment for multiple comparisons
☐	☒	A full description of the statistical parameters including central tendency (e.g. means) or other basic estimates (e.g. regression coefficient) AND variation (e.g. standard deviation) or associated estimates of uncertainty (e.g. confidence intervals)
☐	☒	For null hypothesis testing, the test statistic (e.g. *F*, *t*, *r*) with confidence intervals, effect sizes, degrees of freedom and *P* value noted<br>*Give P values as exact values whenever suitable.*
☒	☐	For Bayesian analysis, information on the choice of priors and Markov chain Monte Carlo settings
☒	☐	For hierarchical and complex designs, identification of the appropriate level for tests and full reporting of outcomes
☒	☐	Estimates of effect sizes (e.g. Cohen's *d*, Pearson's *r*), indicating how they were calculated

Our web collection on statistics for biologists contains articles on many of the points above.

Software and code

Policy information about availability of computer code

Data collection	CytExpert v2.4 (Beckman Coulter), Eve v1.8.2 (Nanolive), Image Lab v6.0 (Biorad), SoftMax Pro v7 (Molecular Devices), VisiView v4.0 (Visitron System), ZEN Black v2.3 (ZWISS).
Data analysis	CellProfiler v4.1.3 (Broad Institute), Flow Jo v10 software (Treestar, Inc), GraphPad Prism v9 (GraphPad Software), Image J v1.52 &1.53 (NIH), Masslynx V4.2 (Waters), MetaboAnalyst online platform v5.0 (PMID 31756036), ZEN Blue v3.2 (ZWISS), Topspin 4.0.6 (Bruker).

For manuscripts utilizing custom algorithms or software that are central to the research but not yet described in published literature, software must be made available to editors and reviewers. We strongly encourage code deposition in a community repository (e.g. GitHub). See the Nature Portfolio guidelines for submitting code & software for further information.

Data

Policy information about availability of data

All manuscripts must include a data availability statement. This statement should provide the following information, where applicable:

- Accession codes, unique identifiers, or web links for publicly available datasets
- A description of any restrictions on data availability
- For clinical datasets or third party data, please ensure that the statement adheres to our policy

All data are available in the Article and the Supplementary Information, and from the corresponding author on reasonable request. Gel source images are shown in

Supplementary Fig. 2. All source data are provided in this paper. Cancer cell line data were mined from https://depmap.org/portal/. The prediction for phase separation of FSP1 was conducted by https://iupred2a.elte.hu and https://mobidb.bio.unipd.it.

Research involving human participants, their data, or biological material

Policy information about studies with human participants or human data. See also policy information about sex, gender (identity/presentation), and sexual orientation and race, ethnicity and racism.

Reporting on sex and gender	N/A
Reporting on race, ethnicity, or other socially relevant groupings	N/A
Population characteristics	N/A
Recruitment	N/A
Ethics oversight	N/A

Note that full information on the approval of the study protocol must also be provided in the manuscript.

Field-specific reporting

Please select the one below that is the best fit for your research. If you are not sure, read the appropriate sections before making your selection.

☒ Life sciences ☐ Behavioural & social sciences ☐ Ecological, evolutionary & environmental sciences

For a reference copy of the document with all sections, see nature.com/documents/nr-reporting-summary-flat.pdf

Life sciences study design

All studies must disclose on these points even when the disclosure is negative.

Sample size	For in vitro experiments, sample sizes were determined based on previous similar studies that have given statistically significant results (PMID: 31634899, 35922516). The number of animals studied per treatment group was determined based on our preliminary data and previous similar studies that have given statistically significant results (PMID: 35922516 and 25402683), and respects the limited use of animal models in line with the 3R recommendations: Replacement, Reduction, Refinement.
Data exclusions	No data exclusions.
Replication	The experimental findings were reproduced as validated by at least three independent experiment in Fig 1c-f, Fig 2b-e, Fig 3a-e, Fig 4b-c and e, Extended Fig 2a-c and i, Extended Fig 3a-k; and at least two independent experiments in Fig 1b and g, Fig 4f-h, Extended Fig 1b-i,k and l, Extended Fig 2d-h and j-l, Extended Fig 3l-p, Extended Fig 4a-h, Extended Fig 5, Extended Fig 6, Extended Fig 7c and e-h, Extended Fig 8c-j, Extended Fig 9a-c and f-h, and Extended Fig 10d-e.
Randomization	For animal studies, mice were randomized into separate cages. For in vitro studies, randomization is not relevant since cells come in millions of populations and are automatically randomized and seeded to different wells for treatment.
Blinding	For animal study, mice were given a number prior to data collection and analysis. Data was collected and analyzed blindly. For in vitro experiments, investigators were not blinded, as standard in this manner of study, which contained multiple steps requiring distinct operations for accuracy and precision precluding blinding to experimental variables.

Reporting for specific materials, systems and methods

We require information from authors about some types of materials, experimental systems and methods used in many studies. Here, indicate whether each material, system or method listed is relevant to your study. If you are not sure if a list item applies to your research, read the appropriate section before selecting a response.

Materials & experimental systems

n/a	Involved in the study	
☐	☒	Antibodies
☐	☒	Eukaryotic cell lines
☒	☐	Palaeontology and archaeology
☐	☒	Animals and other organisms
☒	☐	Clinical data
☒	☐	Dual use research of concern
☒	☐	Plants

Methods

n/a	Involved in the study	
☒	☐	ChIP-seq
☐	☒	Flow cytometry
☒	☐	MRI-based neuroimaging

Antibodies

Antibodies used

GPX4 (1:1000 for WB, ab125066, Abcam), 4-HNE (0.5 g/mL for IF, MHN-20P, JaICA), human FSP1 (1:1000, sc-377120, Santa Cruz Biotechnology), human FSP1(1:5 for WB, clone AIFM2 6D8, rat IgG2a, developed in-house: available from Sigma, Cat#MABC1638-25UL), mouse FSP1 (1:100 for WB, clone AIFM2 1A1 rat IgG2a, developed in-house), mouse FSP1(1:5 for WB and undilute or 1:1 for ICC and IF, clone AIFM2 14D7 IgG2b, developed in-house), human SLC7A11 (1:10; Rat IgG2a monoclonal antibody against an N-terminal peptide of hXCT, clone 3A12-1-1, developed in-house) mouse SLC7A11 (1:1000, Cell Signaling Technology, Cat#98051S), ACSL4 (1:1000, clone A-5, Santa Cruz, Cat#sc-271800), β-actin-HRP (1:50000, A3854, Sigma-Aldrich), valosin containing protein (VCP, 1:10000, ab11433 or ab109240, Abcam), Calnexin (1:100 for ICC, ab22595, Abcam), GM130 (1:100 for ICC, Clone: EP892Y, ab52649, Abcam), EEA1 (1:100 for ICC, Clone: C45B10, 3288S, Cell SignalingTechnology), and LAMP1 (1:100 for ICC, Clone: H4A3, sc20011, Santa Cruz Biotechnology), HA tag (YPYDVPDYA, 1:1000 for WB and 1:10 for IF, clone 3F10 rat IgG1, developed in-house), FLAG tag (DYKDDDDK, 1:5000, 2368S, Cell Signaling Technology), Goat anti-Rat Alexa Fluor 488 IgG (H+L) (1:500, A-11006, Invitrogen), Goat Anti-Mouse IgG H&L Alexa Fluor 647 (1:500, ab150115, Abcam), Donkey anti-rat IgG alexa fluor 555 (1:500, ab150154, Abcam) were used in this study.

Validation

GPX4 (ab125066), VCP (ab11433 or ab109240), Human FSP1 (sc-377120), HA tag (clone 3F10), FLAG tag (2368S), β-actin-HRP (A3854), ACSL4 (sc-271800), human SLC7A11 (clone 3A12) antibodies were validated for WB using mouse and human cells samples in previous publications (PMID: 35922516, 31634899, and 27842070).
4-HNE (MHN-20P) antibody was validated for IHC using mouse samples in a previous publication (PMID: 35922516) and for IF in this study in Extended Fig 8d,h.
FSP1 antibody (clone AIFM2 1A1/6D8 rat IgG2a, and clone AIFM2 14D7 IgG2b, developed in-house) has been validated for WB in previous study (PMID: 35922516), and for ICC/IF in this study in Extended Fig 4d.
Calnexin antibody (ab22595) was validated for ICC using human and mouse cell samples on Cell Signaling Technology website.
GM130 antibody (ab52649) was validated for WB using mouse embryonic fibroblasts on Abcam website and for ICC in this study in Extended Fig 4b.
mouse xCT/SLC7A11 antibody (98051S) was validated for WB using mouse cell samples on Cell Signaling Technology website.
EEA1 antibody (3288S) was validated for ICC using human and mouse cell samples on Cell Signaling Technology website.
LAMP1 antibody (sc20011) was validated for ICC using human and mouse cell samples on Santa Cruz Biotechnology website.
Secondary antibodies were validated for ICC/IF on each provider on website (Cell Signaling Technology, Invitrogen, Abcam).

Eukaryotic cell lines

Policy information about cell lines and Sex and Gender in Research

Cell line source(s)

4-OH-TAM-inducible Gpx4-/- murine immortalized fibroblasts (Pfa1) were reported previously (PMID: 18762024). HT-1080 (CCL-121), 786-O (CRL-1932), A375 (CRL-1619), B16F10 (CRL-6475), HEK293T (CRL-3216), HT-29 (HTB-38), NCI-H460 (HTB-177), MDA-MB-436 (HTB-130), and 4T1 (CRL-2539) cells were obtained from ATCC. THP-1 cells were obtained from DSMZ (Germany). Human PBMC cells were purchased from Tebu-bio (Cat#088SER-PBMC-F). SUDHL5, SUDHL6, DOHH2, and OCI-Ly19 cells (available from DSMZ) were kindly gifted from Dr. Stephan Hailfinger. Rat1 cells (available from Thermo Fisher) were kindly gifted from Medizinische Hochschule Hannover.

Authentication

None of the cell lines used were authenticated.

Mycoplasma contamination

All cell lines were tested negative for mycoplasma contamination.

Commonly misidentified lines
(See ICLAC register)

No commonly misidentified cell lines were used.

Animals and other research organisms

Policy information about studies involving animals; ARRIVE guidelines recommended for reporting animal research, and Sex and Gender in Research

Laboratory animals

Five to six-weeks-old female C57BL6/J and athymic nude mice were obtained from Charles River and six to seven weeks-old mice were used for experiments. All mice were kept under standard conditions with water and food ad libitum and in a controlled environment (22 ± 2°C, 55 ± 5% humidity, 12 h light/dark cycle) in Helmholtz Munich animal facility under SPF-IVC standard conditions.

Wild animals	The study did not involve wild animals.
Reporting on sex	Experiments were performed on only female mice.
Field-collected samples	The study did not involve field-collected samples.
Ethics oversight	All experiments were performed in compliance with the German Animal Welfare Law and have been approved by the institutional committee on animal experimentation and the government of Upper Bavaria (ROB-55.2-2532.Vet_02-17-167).

Note that full information on the approval of the study protocol must also be provided in the manuscript.

Flow Cytometry

Plots

Confirm that:

☒ The axis labels state the marker and fluorochrome used (e.g. CD4-FITC).

☒ The axis scales are clearly visible. Include numbers along axes only for bottom left plot of group (a 'group' is an analysis of identical markers).

☒ All plots are contour plots with outliers or pseudocolor plots.

☒ A numerical value for number of cells or percentage (with statistics) is provided.

Methodology

Sample preparation	100,000 cells per well were seeded on a 12-well plate one day prior to the experiments. On the next day, cells were treated with 2.5 µM icFSP1 for 3 h, and then incubated with 1.5 µM C11-BODIPY 581/591 (Invitrogen, Cat#D3861) for 30 min in a 5% CO2 atmosphere at 37°C. Subsequently, cells were washed by PBS once and trypsinized, and then resuspended in 500 µL PBS. Cells were passed through a 40 m cell strainer and analyzed by a flow cytometer (CytoFLEX, Beckman Coulter) with a 488-nm laser for excitation. Data was collected from the FITC detector (for the oxidized form of BODIPY) with a 525/40nm bandpass filter and from the PE detector (for the reduced form of BODIPY) with a 585/42 nm bandpass filter.
Instrument	CytoFLEX (Beckman Coulter)
Software	CytExpert v2.4 was used for data collection. FlowJo v10 was used for data analysis.
Cell population abundance	At least 10,000 cells were analyzed for each sample.
Gating strategy	Cell populations were separated from cellular debris using FSC and SSC.

☒ Tick this box to confirm that a figure exemplifying the gating strategy is provided in the Supplementary Information.
